# Interaction between Coastal and Oceanic Ecosystems of the Western and Central Pacific Ocean through Predator-Prey Relationship Studies

**DOI:** 10.1371/journal.pone.0036701

**Published:** 2012-05-15

**Authors:** Valerie Allain, Emilie Fernandez, Simon D. Hoyle, Sylvain Caillot, Jesus Jurado-Molina, Serge Andréfouët, Simon J. Nicol

**Affiliations:** 1 Oceanic Fisheries Programme, Secretariat of the Pacific Community, Noumea, New Caledonia; 2 Unité de Recherche 227– CoRéUs, Institut de Recherche pour le Développement, Noumea, New Caledonia; Institute of Marine Research, Norway

## Abstract

The Western and Central Pacific Ocean sustains the highest tuna production in the world. This province is also characterized by many islands and a complex bathymetry that induces specific current circulation patterns with the potential to create a high degree of interaction between coastal and oceanic ecosystems. Based on a large dataset of oceanic predator stomach contents, our study used generalized linear models to explore the coastal-oceanic system interaction by analyzing predator-prey relationship. We show that reef organisms are a frequent prey of oceanic predators. Predator species such as albacore (*Thunnus alalunga*) and yellowfin tuna (*Thunnus albacares*) frequently consume reef prey with higher probability of consumption closer to land and in the western part of the Pacific Ocean. For surface-caught-predators consuming reef prey, this prey type represents about one third of the diet of predators smaller than 50 cm. The proportion decreases with increasing fish size. For predators caught at depth and consuming reef prey, the proportion varies with predator species but generally represents less than 10%. The annual consumption of reef prey by the yellowfin tuna population was estimated at 0.8±0.40CV million tonnes or 2.17×10^12^±0.40CV individuals. This represents 6.1%±0.17CV in weight of their diet. Our analyses identify some of the patterns of coastal-oceanic ecosystem interactions at a large scale and provides an estimate of annual consumption of reef prey by oceanic predators.

## Introduction

The tropical area of the Western and Central Pacific Ocean (WCPO) represents a vast area of about 35 million km^2^ (120°E-140°W, 15°N-25°S), larger than the Indian and Atlantic Ocean tropical areas. Compared to these oceans and to the Eastern Pacific, the WCPO is uniquely scattered with many atolls, high islands and island groups [1], totaling about 140,000 km of coast (excluding Australia) with diverse habitats including lagoons and reefs. The WCPO is also characterized by complex bathymetry with numerous seamounts [2], [3]. This unique topography induces multiple and complex vertical hydrological structures and current circulation patterns (eddies, frontal zones) [1].

The WCPO region is also characterized by tuna fisheries that generate the highest tuna catches in the world (>60% of the global tuna catches) with skipjack, yellowfin, bigeye and albacore tuna annual catches estimated at nearly 2.5 million tonnes in recent years [4]. In 2011 the total estimated landed value of tuna catches in this region exceeded USD 4 billion [4] representing a major economic resource for Pacific Island Countries and Territories [5].

The complex structure of the WCPO coastal system and its spread over such a large area where important oceanic fisheries operate create the opportunity for a high degree of interaction between coastal and oceanic ecosystems. Organisms with an obligate coastal, reef or lagoon life-history phase, named reef prey in our study, drifting in the oceanic domain before coming back to the reef, have a role in transferring energy between the coastal and the oceanic realm and vice versa.

Interactions between coastal and oceanic ecosystems have been explored through predator-prey relationship studies. Reef organisms have been identified as prey of the oceanic predators in a number of diet analyses of large pelagic fish [6]–[22]. According to the studies considered, their importance in the diet in terms of frequency and quantity varied from minor [6], [8], [15], [22] to major or dominant [9]–[11], [14], [16]–[20]. Proportion in the diet varied according to factors such as the predator species considered (surface feeders tend to consume more reef prey) [7], [10], [18], [21], size of the predators (large fish eat less reef prey) [11], [17], and habitats (e.g. near-shore predators eat more reef prey than offshore fish) [9]–[10], [13]–[14], [16]–[18].

However, most of these studies were based on limited sample size (fewer than 400) collected in restricted areas, in the open ocean less than 100 km from land, close to land or around near-shore fish aggregating devices (FAD) anchored in deep waters. These studies do not offer the possibility to systematically analyze the influence of multiple factors such as distance to land or reef, predator species, or predator length, latitude and longitude so as to properly characterize reef prey consumption by oceanic predators at a large scale. Moreover, to our knowledge, the total amount of reef prey consumed by predator’s populations has never been estimated.

Trophic studies conducted by the Secretariat of the Pacific Community (SPC) in the whole WCPO provide a unique opportunity to explore the potential importance of reef prey for the offshore pelagic ecosystem at an ocean basin scale. We examine patterns of interaction between coastal ecosystems and oceanic ecosystems in the WCPO by applying generalized linear models (GLM) to this large dataset of oceanic predator stomach contents data, and present an example of estimating total annual consumption of reef prey by oceanic predators in the WCPO.

## Results

### 1. Description of Reef Prey Consumed

The most important reef prey found in the stomach contents were Acanthuridae (surgeonfish), Balistidae (triggerfish), Chaetodontidae (butterflyfish), Holocentridae (squirrelfish), Monacanthidae (filefish), Pomacanthidae (angelfish), Siganidae (rabbitfish), Synodontidae (lizardfish) larval and juvenile fishes, and Stomatopoda (mantis shrimp), Brachyuran (crabs) and Palinuroidea (lobsters) crustacean larvae (Table S1). Average standard length (SL) and weight (±SE) of larval and juvenile fishes were 32.9±0.4 mm and 1.85±0.14 g (30, 24, 20, 35, 36, 15, 52 mm and 1.26, 1.25, 0.60, 1.59, 1.78, 0.24, 1.88 g respectively for families cited previously except Synodontidae for which no individual measures were available) and average cephalo-thorax length and weight for crustaceans larvae were 7.5±0.2 mm and 0.27±0.02 g (8.4, 4.9 and 14.5 mm and 0.25, 0.13 and 1.13 g respectively for groups cited previously).

### 2. Probability of Consumption or Frequency of Occurrence of Reef Prey by Large Predators

**Figure 1:**
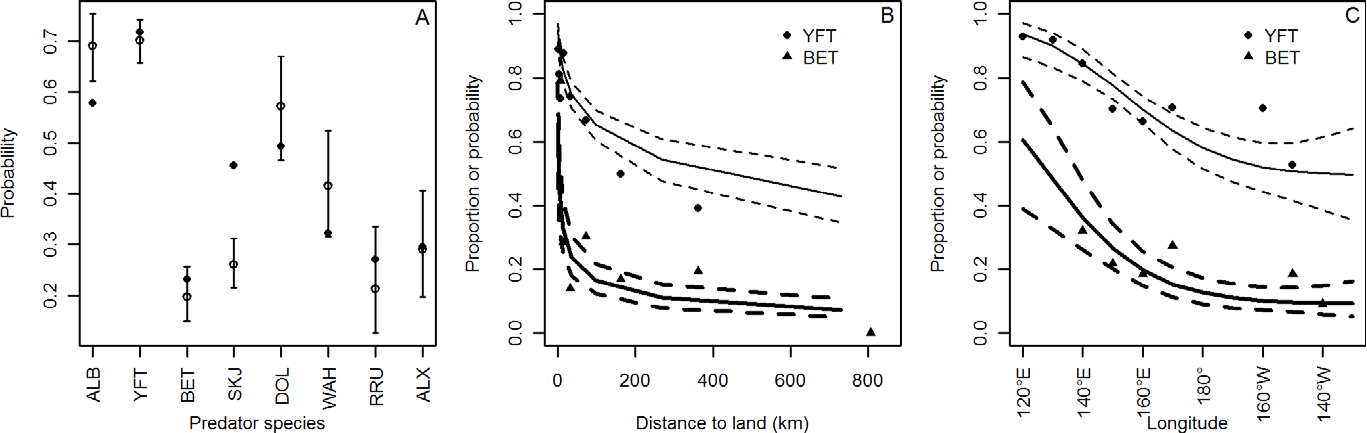
Observed proportion (frequency of occurrence) and model predicted probability with 95% confidence interval of the number of stomachs containing reef prey for all predators. A) By predator species, B) by distance-to-land and C) by longitude. Solid dots are observations, open circles with error bars or solid and dashed lines are predicted probabilities and 95% confidence intervals. YFT (solid circles and normal lines) and BET (triangles and bold lines) are shown as examples in B) and C). One variable was predicted at a time from the results of the model by fixing the other variables at median value. In Figure 1A, because the predictions are established for median values of distance-to-land and longitude, some discrepancies between predicted and observed values are apparent, particularly for SKJ, as observed data come from places on average significantly different from median values chosen for predictions. ALB, albacore tuna; YFT, yellowfin tuna; BET, bigeye tuna; SKJ, skipjack; DOL, dolphinfish; WAH, wahoo; RRU, rainbow runner; ALX, lancetfish.

The chosen model for explaining the reef prey occurrence in stomach contents included predator species, distance-to-land and longitude (Table 1, Table S2). At the median longitude and distance-from-land, albacore and yellowfin tuna had the highest probabilities of consuming reef prey (>0.6), followed by skipjack, mahi mahi and wahoo which had intermediate probabilities (>0.3 and <0.6), while bigeye, rainbow runner and lancetfish had lower probabilities (<0.3) of consuming reef prey (Figure 1*A*). The highest proportions of stomachs containing reef prey were observed closer to land and decreased logarithmically as distance-to-land increased (Figure 1*B*). The probability that a stomach contained reef prey decreased strongly in the first 100 km from land. The data indicated a clear spatial pattern with higher probabilities of presence of reef prey in the stomach of predators located in the western part of the area (Figure 1*C*). The probability decreased towards the date line (180°) and stabilized at lower values east of the dateline up to the eastern limit of our study area (130°W).

**Table 1:** Results of GLM modeling presence-absence of reef prey in stomach contents of predators.

	Df	Chisq	p-value	BIC
set_code random effect				5123
+ predator	7	398.7	<2.2e-16 ***	4745
+ log(dist_land+1)	1	61.4	4.8e-15 ***	4667
+ ns(longitude, df = 2)	2	40.8	1.4e-09 ***	4645

Df, degree of freedom; Chisq, Deviance of the final model; p-value from Anova Chi-test; BIC, Bayesian Information Criterion. Significance codes: 0 ‘***’ 0.001 ‘**’ 0.01 ‘*’ 0.05 ‘.’ 0.1 ‘ ’ 1.

### 3. Weight Proportion of Reef Prey in Predator’s Stomach Consuming Reef Prey

**Figure 2:**
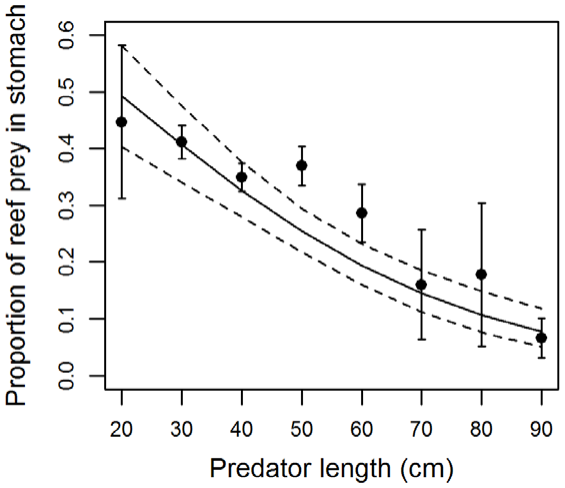
Proportion by weight of reef prey in stomach content against predator’s length, the main explanatory variable, for all predators consuming reef prey and caught with surface fishing gear. Observed mean (dot) with 95% confidence interval (error bars) and predicted value (solid line) with 95% confidence interval (dashed line).

**Figure 3:**
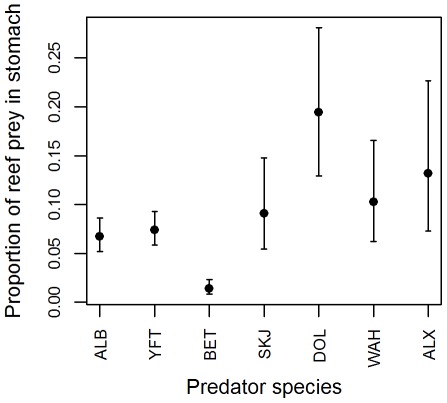
Proportion by weight of reef prey in stomach content against predator’s species, the main explanatory variable, for all predators consuming reef prey and caught with longline gear. Predicted means with 95% confidence interval. Predator code: see caption of figure 1. No rainbow runner (RRU) was caught with longline gear.

The preferred model for explaining the weight proportion of reef prey in the stomach of the predator consuming reef prey only included fishing gear (Table S3). Predators caught with surface gears contained a higher proportion of reef prey in their stomachs than predators caught with longline gear: 0.27±0.01 vs. 0.07±0.04 respectively (predicted mean and 95% confidence interval). However, samples collected with these two fishing gear types were different in terms of predator species, length, and longitude. Further modeling was conducted separately on surface and longline gears to identify additional determining factors. The preferred model when only considering samples from predators caught by surface gears included the length of the predator (Table S4). The weight proportion of reef prey per stomach was more than 0.3 for small predators (20 to 40–50 cm) and the proportion decreased with the size of the predator (Figure 2). The preferred model when only considering samples from predators caught by longline gears included only predator species (Table S5). Bigeye tuna consumed the lowest weight proportion of reef prey with less than 0.02 (Figure 3). Albacore and yellowfin consumed similar proportions, predicted to be between 0.05 and 0.1. Predicted weight proportions were between 0.09 and 0.19 for other predators (Figure 3), however their confidence intervals were large, most likely due to the small sample size (<50) for these predators.

### 4. Reef Prey Consumption Estimate

The yellowfin tuna population, estimated at 1.47 million tonnes in 2009 in the equatorial Western and Central Pacific Ocean between 20°N and 10°S [23], consumed an annual estimate of 0.818±0.40CV million tonnes of reef prey, representing 6.1%±0.17CV of the 13.426±0.36CV million tonnes of preys consumed by this predator. Based on average weight of reef prey crustaceans and fish (cf Results §1) and on reef prey proportions in weight of crustaceans (0.67±0.02CV) and fish (0.33±0.04CV) consumed by yellowfin tuna, the total number of reef prey consumed by the yellowfin tuna population in the WCPO was estimated to be 2.17×10^12^±0.40CV individuals (2.02×10^12^±0.41CV crustaceans and 0.15×10^12^±0.41CV fishes).

## Discussion

Our analyses suggest that reef organisms are a frequent prey for small oceanic predators in the WCPO. They also suggest that the degree of interaction is affected by the spatial distribution of reef prey: the majority of reef prey is consumed in the western part of the region and at distance-to-land less than 100 km. The predator’s behavior also influences the reef prey consumption as small specimens and species foraging at the surface eat more reef prey. A total of 0.8 million tonnes of reef prey (about 2.2×10^12^ specimens) is estimated to be consumed by the yellowfin tuna population in the region, representing 6.1% in weight of their diet.

We observed that more interaction occurs west of 160°E in the area that encompasses the Western Pacific warm pool ecosystem (e.g. Indonesia, Palau, Papua New Guinea, Federated States of Micronesia and Solomon Islands). The decreasing trend observed from west to east can be explained by the topography and its related oceanography. The western region of the WCPO has more islands, more coast line and higher reef surface area than the eastern region of the studied area (Figure S1). In general the production of reef larvae is positively correlated with the quantity of reef and coastal habitats [24]–[25] and consequently higher availability of reef prey can be expected in the western region than in the eastern region of our study area. The pelagic phase of reef larvae can extend up to 1 year [26] for some species and it has been demonstrated that the longer the duration of the pelagic larval phase, the wider the potential dispersion [27]. Duration of the pelagic phase along with dispersion is one of the most important factors influencing the distribution and availability of reef prey and consequently their presence in stomach contents. Late-stage reef larvae are effective swimmers [28]–[29], but before developing these capabilities, part of the reef prey is assumed to be advected by currents [30]. Large-scale circulation patterns will tend to disperse larval organisms from their spawning site by several hundred kilometers [31]–[32]. Complex topography with many islands and seamounts disrupts the flow of the main currents and induces the formation of eddies in the lee of the islands in the WCPO [33]–[34]. These eddies and other oceanographic features act as strong larval retention zones [26], [31], [35]–[36]. We observed an association between reef prey presence in predator stomachs and the proximity to land, with a high probability that reef prey are consumed within 0–100 km of land. This trend has been previously noted in other tuna stomach content studies [7], [13], [17]–[18] and in fish larvae distribution studies [37], [38]. This association is in agreement with larval retention zones occurring around islands. In our model, at the scale of the ocean basin, the proportion of reef prey in stomachs was better explained by proximity to land than proximity to reef which might be linked to the prevalent effect of larval retention associated to land masses over the production effect of reefs. We acknowledge that, at different scales, the results of the model might differ. Reef could be a better predictor than land in areas dominated by atolls such as Tuamotu Archipelago in Central Pacific, as atolls and banks may be characterized by large reef and lagoon areas without land. Conversely, several large islands do not necessarily have reefs around them (Marquesas Islands, Vanuatu Islands). Surface of reef or lagoon around the predator were not included in the model but they are expected to be positively correlated with presence and proportion of reef prey in the stomachs if it is assumed that a larger surface of reef produces more reef prey.

The school type (FAD vs. non-FAD school) did not appear as a significant explanatory variable in our chosen models. However it came forward in models ranked within the 5 best models with lowest BIC (Table S2, Table S3, Table S4). In the WCPO there are large arrays of anchored FADs in Papua-New-Guinea and Solomon Islands particularly and many drifting FADs in the western part of the region. In this region the anchored and drifting FADs are located offshore. Predators were caught on average on anchored FADs, drifting FADs and drifting logs located respectively at 68±6 km (mean±95% confidence interval), 152±17 km and 103±22 km from shore while fish from free schools were caught at 147±22 km from shore. These distances are much larger than in other studies on the impact of FADs on the diet of oceanic predators which are dealing with near-shore anchored FADs less than 30 km from shore [9]–[11], [14], [16]. Like floating sargassum, FADs do have associated fauna which is largely composed of reef pre-settlement larvae and juveniles [39]–[42]. Many studies have suggested that oceanic predators caught in the vicinity of FADs or floating sargassum contained a large proportion of reef prey [10], [14], [16], [43]–[44]. One study is discordant however as Brock observed that, around Hawaii, reef prey were dominant in non-FAD predators while FAD predator were mainly feeding on deep crustaceans [9]. Results of this particular study might be linked to a specific availability of this shrimp. If our study indicates that FAD associated predators also consumed reef prey, it is not a major explanatory variable in our models because their large distance from shore probably means they aggregate less reef prey than near-shore FADs. It could also be linked to the scale of our study which includes confounding factors: most of the anchored FADs are located in the western part of the region. Studies at smaller spatial scale might reveal the prominence of the FAD effect on the diet.

In the water column reef prey are commonly distributed in the upper 100 to 200 m, with maximum abundance observed between 10 and 100 m for fish and crustaceans larvae [26], [31], [35]. This vertical distribution matches our observation that predators captured with surface gear consume a higher proportion of reef prey than predators captured at depth by longline. Large oceanic fishes and particularly tuna are considered to be opportunistic predators feeding on any available prey [45]–[46], but access to this prey depend upon the habitat preferences of predators, particularly the depth range linked to diving possibilities, temperature and oxygen tolerances [47]–[48]. Bigeye and lancetfish for example are deep dwellers [49] and will therefore have limited interactions with surface reef prey. Both species show low probability and low proportion of reef prey in their diet as also noted in other studies [7], [15], [21]. On the other hand, yellowfin, skipjack, dolphinfish and wahoo forage mainly at the surface [48], [50] and, in our study as well as in others [7], [21], they show higher consumption of reef prey. Albacore appears to be a special case as our data indicated they frequently consume reef prey despite the adults being considered deep dwellers [51]–[52]. Albacore show a pronounced preference for crustaceans in their diet [7], and in our study they consumed reef crustaceans, crab (Brachyuran), mantis shrimp (Stomatopoda) and lobster larvae (Palinura), more frequently and in larger quantities than reef fish larvae. The forage biology of albacore is highly uncertain, but diel vertical migrations that are common to many tuna species have been observed in albacore, where they migrate to shallower habitats at night and deeper habitats during the day [53].

The size ratios between predator and prey also influence the consumption of reef prey. Our analyses suggested that the diet contribution of reef prey, smaller than non-reef prey on average (35 vs. 78 mm SL for fish prey), was higher for predators smaller than 40–50 cm caught at the surface than for larger predators. Graham [11] and Nakamura [17] also observed this trend mainly due to a decrease of the consumption of reef crustaceans’ larvae when predator’s size increased. In Graham’s study [11], the consumption of reef fish larvae increased with the size of the predator but did not compensate the decrease of crustaceans inducing an overall decrease of reef prey. We also found that very large species such as shark and billfish did not consume reef prey. This observed higher consumption of small reef prey by small predators matches the previously observed trend of increased mean prey size with increasing predator size in fish communities [46], [ 54]–[55].

The estimated annual amount of prey (reef and oceanic) consumed by the yellowfin population in the WCPO (13.4 million tonnes or 0.39 tonnes/y/km^2^) is in the same order than previous estimates for yellowfin tuna in the same region [56] (10.7 million tonnes or 0.31 tonnes/y/km^2^), in the Eastern Pacific Ocean [57] (0.25 tonnes/y/km^2^), for *Thunnus tonggol* in Australian waters [58] (0.37 tonnes/y/km^2^) and for *Euthynnus affinis* in eastern Australia [59] (0.15 tonnes/y/km^2^). To our knowledge, our study provided for the first time an estimate of reef prey consumed by oceanic fish predation (0.818 million tonnes±0.4CV- 2.17×10^12^±0.40CV individuals –6.1%±0.17CV in yellowfin tuna population diet) which indicate their importance in the diet of top predators and highlight the role of pelagic predators on mortality of coastal organisms during their pelagic offshore phase. However it is difficult to estimate if the impact of this type of predation on the recruitment rate of reef larvae to the reef. Moreover it likely does not have influence on abundance of juvenile and adult reef organisms at the reef as it was suggested that post-recruitment mortality had much greater effect on abundance than recruitment rate [60].

Our study showed the important interactions between coastal and oceanic domains in the WCPO; the question of reciprocal subsidies between these 2 ecosystems remains unanswered. According to our study 0.8 million tonnes of reef prey are consumed by the yellowfin tuna population alone. However this biomass cannot be considered as direct subsidy from coastal to oceanic ecosystem as reef prey left the reefs and lagoons as eggs or at a very early life-stage representing a very small biomass. The energy necessary for eggs and small larvae to develop up to a late larval stage and juvenile consumed by oceanic predators was taken from the oceanic environment. Hence most of the biomass of reef prey consumed by pelagic predators comes from the oceanic ecosystem itself and is not subsidized by the coastal ecosystem. In return the coastal ecosystem is benefiting from the biomass of juvenile reef fish produced in the oceanic ecosystem and coming back to the reef. However it is not possible to estimate how much the oceanic ecosystem is subsidizing the coastal system as it is very difficult to estimate the amount of reef prey coming back to the reef. Their survival once at the reef is poor [60] which will have an impact on their adult’s population abundance, however by being consumed by predators on the reef they do enter the coastal food web and contribute to the coastal ecosystem.

Exploration of multiple models highlighted a specific issue linked to the opportunistic sampling programme used in this study. Our sampling programme relied on fishing operations with surface and longline fisheries, which operate in very different ways and catch fish of different size (small versus large respectively), different species (skipjack versus albacore for example), different school types (FAD and non-FAD versus non-FAD) and different locations (equator versus subtropical), with limited overlap between these factors. Location, FAD schools and distance-to-land are also related parameters as FAD are preferentially anchored in the western part of the region (Papua New Guinea and Solomon Islands). Despite the number of stomachs examined, the large number of co-variates makes it difficult to explore the influence of each covariate independently as the degrees of freedom in the analyses were insufficient to explore all relationships. Close relationships between some of the parameters are apparent when testing all the possible models, as the 4 or 5 best models usually highlighted the importance of related factors (Tables S2, Table S3, Table S4, Table S5). We chose the best model based on the BIC which identified clear trends in the dataset; however some of the parameters not selected in the final model also had support in other models. Moreover, the distribution of the samples through time did not permit exploration of annual and seasonal changes, but temporal variability is likely to be important in our area which undergoes strong interannual variation such as El Niño Southern Oscillation (ENSO) and perceptible seasonality in the subtropical areas. Further analyses are also needed to quantify the contribution of reef prey to the total energy budget of oceanic ecosystem to fully understand the subsidy provided by coastal ecosystems. Nevertheless our analyses based on a robust statistical method, applied to a large dataset and covering a vast area clearly identify some of the patterns in the relationship between coastal and oceanic ecosystems at a large scale and gives for the first time an estimate of the annual consumption of reef prey by oceanic predators.

## Materials and Methods

### Sampling Programme

**Figure 4:**
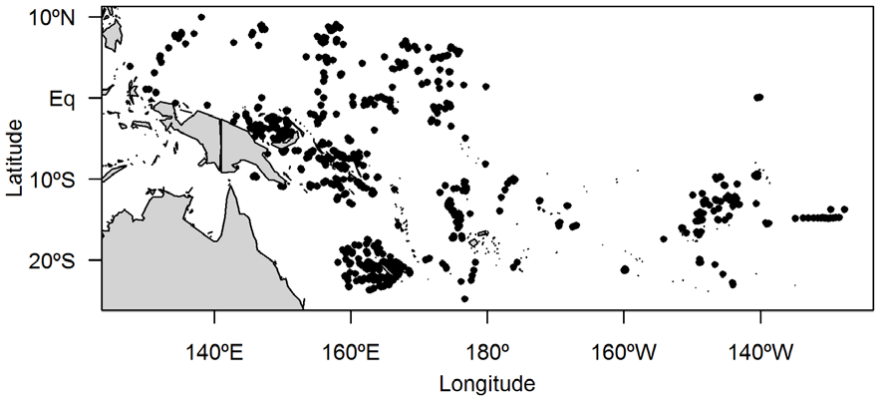
Locations of the 812 sets where samples were collected.

A total of 7633 stomachs of pelagic predators were collected between January 2001 and April 2011 in the Western and Central Pacific Ocean (9°N - 27°S and 127°E - 132°W) (Figure 4). All samples came from commercial fisheries and were already dead when provided to the sampler, no permission was required. Among the 5444 non-empty stomachs, 585 were removed from the dataset due to missing information such as predator length or spatial data. A total of 58 species were sampled with 1 to 1598 non-empty stomachs per species (Table S6). The analysis was conducted on the 4286 non-empty stomachs of the 8 species with more than 100 samples. Samples were collected by fisheries observers and scientists onboard fishing vessels during 812 different sets. Between 1 and 34 predators were collected per set. Fishing gear (longline, purse-seine, pole-and-line, trolling, handline), school association (free school, anchored FAD, drifting FAD, drifting log, seamount, whale and whale shark), predator species, fish length, date, time, position of the catch (position of the beginning of the set for longline gear and trolling for which catch occur when the boat is underway; position of the boat for purse-seine, pole-and-line and handline for which catch occur when the boat is stationary), were recorded on logsheets. Samples were frozen onboard.

### Stomach Content Examination

Stomachs were considered empty when containing only digestive fluids. For non-empty stomachs, prey were identified to the lowest taxonomic level possible. Identification keys used for prey identification were, for fish: Smith & Heemstra [61] and Carpenter & Niem [62], for crustaceans: Poore [63], for cephalopods: Young et al [64], for invertebrates: Wrobel & Mills [65], and for zooplankton: Boltovskoy [66]. For each prey taxon the total weight in grams and the number of specimens were determined; the weight and length of individual specimens were measured when possible according to their digestion stage. Reef or oceanic origin of the prey was determined based on bibliographic information. However, due to their advanced stage of digestion, 22% (in weight) of the prey were not identified to a taxonomic level sufficient to determine their origin. These were grouped with oceanic prey, which were the most numerous; the analysis could therefore underestimate the proportion of reef prey.

### Response Variable

Quantitative analysis of stomach contents can be achieved in different ways [67]. In this study only two prey items were considered: reef prey and oceanic prey, and were expressed as proportions of weights per stomach. Proportion in weight of reef prey was used as the response variable.

### Co-variates

The explanatory variables included in the model were predator’s species (pred_code), predator’s length (pred_L), latitude (lat_dec) and longitude (lon_dec) of catch, gear code (gr_code), school code (sch_code) and distance to closest land (dist_land) or closest reef (dist_reef). Surface of reef or lagoon in a 100-nautical miles-diameter-circle around the predator were also included in preliminary models, however considering that these data were not available for about 20% of the samples, these variables were not included in the model. Considering the opportunistic nature of the sampling, the number of samples per year or month did not allow taking into account temporal variability. Fishing gears were grouped into longline (catching fish from the surface down to approximately 400 meters depth) and surface gears (pole-and-line, trolling, purse-seine, handline, catching fish at the surface). School associations were grouped into FAD (anchored FAD, drifting FAD, drifting log, whale and whale shark) and non-FAD schools (free school, seamounts). Distance between the sampled predator and the closest land was calculated based on predator’s position and land information established by the National Geospatial Intelligence (NGA) World Vector Shoreline (WVS) (http://shoreline.noaa.gov/data/datasheets/wvs.html). Distance between the sampled predator and the closest reef was calculated based on predator’s position and reef data from the Millennium Coral Reef Mapping Project (MCRMP) [68] and the lower resolution United Nations Environment Programme (UNEP) -World Conservation Monitoring Centre (WCMC) Global Coral Reef distribution (2010) (www.unep-wcmc.org) for locations where MCRMP was not available (Fiji, Philippines, Indonesia, and North Papua New Guinea).

### Statistical Models

The frequency distribution of reef prey weight in pelagic predator stomachs exhibits skewness and a spike at zero (2780 zero, i.e. 52% of the values). To account for this data structure we analyzed the data in two parts using a delta generalized linear models (GLM) by modelling occurrence separately from the quantity observed [69]. In the first model, the response variable was defined as presence of reef prey in the sample. These data were analyzed using a generalized linear mixed model fitted using the Laplace approximation, with a binomial response and logit link function, with a random effect applied to fishing set as predators caught in the same set were not considered to be independent. In the second approach only the samples containing reef prey were analyzed using the reef prey by weight as a proportion of the total prey. The statistical distribution of this variable was markedly non-normal. We normalized its distribution by transforming it using a logit function. Samples that contained 100% of reef prey (7% of the predator samples) were omitted (as logit(1) = Inf.). Data were analyzed using a generalized linear mixed model with a Gaussian response and identity link function, with a random effect applied to fishing set. Both analyses used the function lmer in the package lme4 in R version 2.12.1 [70]. In each case, the complete range of models from Model 1 to Model 2 (below) with all the possible combinations of co-variates was explored.

Model1: response variable ∼ (1|set).

Model2: response variable ∼ pred_code+ pred_L+ sch_code+ ns(lon_dec,df = 2)+ ns(lat_dec,df = 2)+ gr_code+ log(dist_land+1) or (dist_reef+1) +(1|set).where (1|set) represents the random effect for fishing set.

Due to their high degree of correlation, co-variates distance-to-land and distance-to-reef were evaluated in separate models. Log transformation and splines with various degrees of freedom were explored for continuous co-variates longitude, latitude, distance-to-land and distance-to-reef. Model fits were compared using the BIC Bayesian Information Criterion [71] and models with lower BIC were preferred. Anova type II tests (Chi-square statistic test) were performed to identify the degree of significance of the covariates.

For the model of occurrence, the chosen model was preferred to the model with the smallest BIC since the BICs were almost equal and the chosen model was simpler with 3 explanatory variables instead of 5 (Table S2). The expected frequency of occurrence or probability-of-consuming-reef-prey *p* by a predator of species *i*, caught at longitude *j* and distance-to-land *k* is defined as follow:



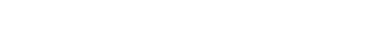
where the alphas are estimated model parameters, the function *f* is a cubic spline estimated with two degrees of freedom and C_1_ is a constant calculated in the model.

Correlation between distance-to-land and longitude was statistically significant (cor = 0.42, p-value <2.2 e^−16^); but including both variables rather than only one significantly improved the model. Despite the correlation these 2 variables affect *p* independently and should be conserved into the model.

For the model of proportion, the preferred model only included fishing gear (Table S3). Further modeling was conducted separately on surface and longline gears (Table S4 and S5). The predicted proportion-of-reef-prey-given-that-reef-prey-was-consumed *q* by a predator of species *i*, of length *m* and caught by fishing gear *g* is defined below:



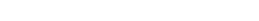
where g indicates purse seine (ps)-caught fish, and



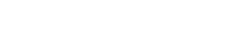
where g indicates longline (ll)-caught fish. The alphas are estimated model parameters and C_2_ and C_3_ are constants calculated in the models.

### Reef Prey Consumption Estimate

The annual consumption of reef prey by yellowfin tuna population in 2009 in the Western and Central Pacific Ocean (110°E to 150°W and 20°N to 10°S) was estimated by combining probability of reef prey occurrence, proportion of reef prey given reef prey was consumed, number of predators and predator’s daily ration. The area was divided into 1/10 degree cells and the longitude and the distance to land of the center of the cell were determined. Probability and proportion were determined by the statistical models as shown above. Numbers of yellowfin tuna at age and per quarter were extracted from the 2011 yellowfin stock assessment for the year 2009 [23]; we assumed the tuna population was evenly distributed throughout the area considered. Daily ration was adapted from the yellowfin daily ration at length determined by Maldeniya [72] using weight and length at age data from the stock assessment [23].

According to model results above and considering yellowfin tuna catches by longline represent only about 12% of the catches in the equatorial area [23] and only concern large specimens consuming minor quantities of reef preys, we took into account only the purse seine model of reef prey proportion to calculate the annual consumption of reef prey by yellowfin tuna population according to the equation:
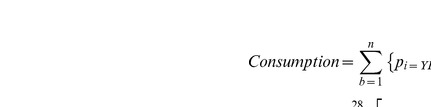
where Consumption is the annual reef prey consumption, *b* represents the number of cells of 1/10 degree square with *n* = 277436, *p_i,j,k_* is the probability of consuming reef prey as defined above by species *i* which is yellowfin (YFT) in this case and for each cell *b* defined by its longitude *j* and the distance-to-land *k* of its center, *a* represents the predator age class (1 to 28 quarters), *q_g,m_* is the proportion of reef prey consumed given that reef prey was consumed as defined above with gear *g* equals to purse seine (ps) and *m* length of the fish, *t* represents the quarter of the year, N is the number of yellowfin tuna of age *a* in cell *b* at quarter *t*, *R* is the predator daily ration at age *a* expressed in proportion of predator’s weight, W is the average weight of the predator at age a.

Uncertainty around the annual consumption estimate was calculated using a randomization method (n = 1000) to combine the uncertainty estimates for each of the main input values. Uncertainties around the probability and proportion estimates were determined from the statistical models detailed in results. For the daily ration no uncertainty was provided by Maldeniya [72], so a coefficient of variation of 20% was assumed. In making this judgment we considered several alternative estimates of daily ration [57], [73]–[74], which were similar to Maldeniya. Uncertainty in the number of predators was determined by aggregating three sources of uncertainty. The stock assessment [23] estimated parameter uncertainty in biomass of 7%, and structural uncertainty of 18%. These are minimum estimates since they are based on assuming that the model is correct. Other factors that cannot be estimated, such as the fact that biomass is not evenly distributed throughout the area, were assumed to contribute substantial additional uncertainty, leading to a summary CV of 30%. Uncertainties around the average weight of individual reef crustacean and fish preys, and around the proportion of crustaceans and fish in yellowfin tuna diet was based on diet data used in this study.

## Supporting Information

Figure S1 Quantification of island and reef coverage per longitudinal band in the western and central Pacific. Within the 15°N to the 25°S latitudinal band and excluding Australia.
(TIF)

Table S1 **Species composition, raw value and percentages of the cumulated weight (W), number (N) and frequency (F) of the reef preys in the diet of the main pelagic predators.**
(DOCX)

Table S2 **Results of the five second-best models of reef prey occurrence in stomach content.**
(DOCX)

Table S3 **Results of the five best models of reef prey proportion in stomach containing reef prey.**
(DOCX)

Table S4 **Results of the five best models of reef prey proportion in stomach containing reef prey for predators caught with surface fishing gears.**
(DOCX)

Table S5 **Results of the five best models of reef prey proportion in stomach containing reef prey for predators caught with longline fishing gear.**
(DOCX)

Table S6 **Number of non-empty stomachs examined for the 58 species collected.**
(DOCX)
