# Supplementary figures and images for: Interaction between Coastal and Oceanic Ecosystems of the Western and Central Pacific Ocean through Predator-Prey Relationship Studies
Source: PLoS One. 2012 May 15;7(5):e36701. doi: 10.1371/journal.pone.0036701 (PMC3352925; doi:10.1371/journal.pone.0036701)

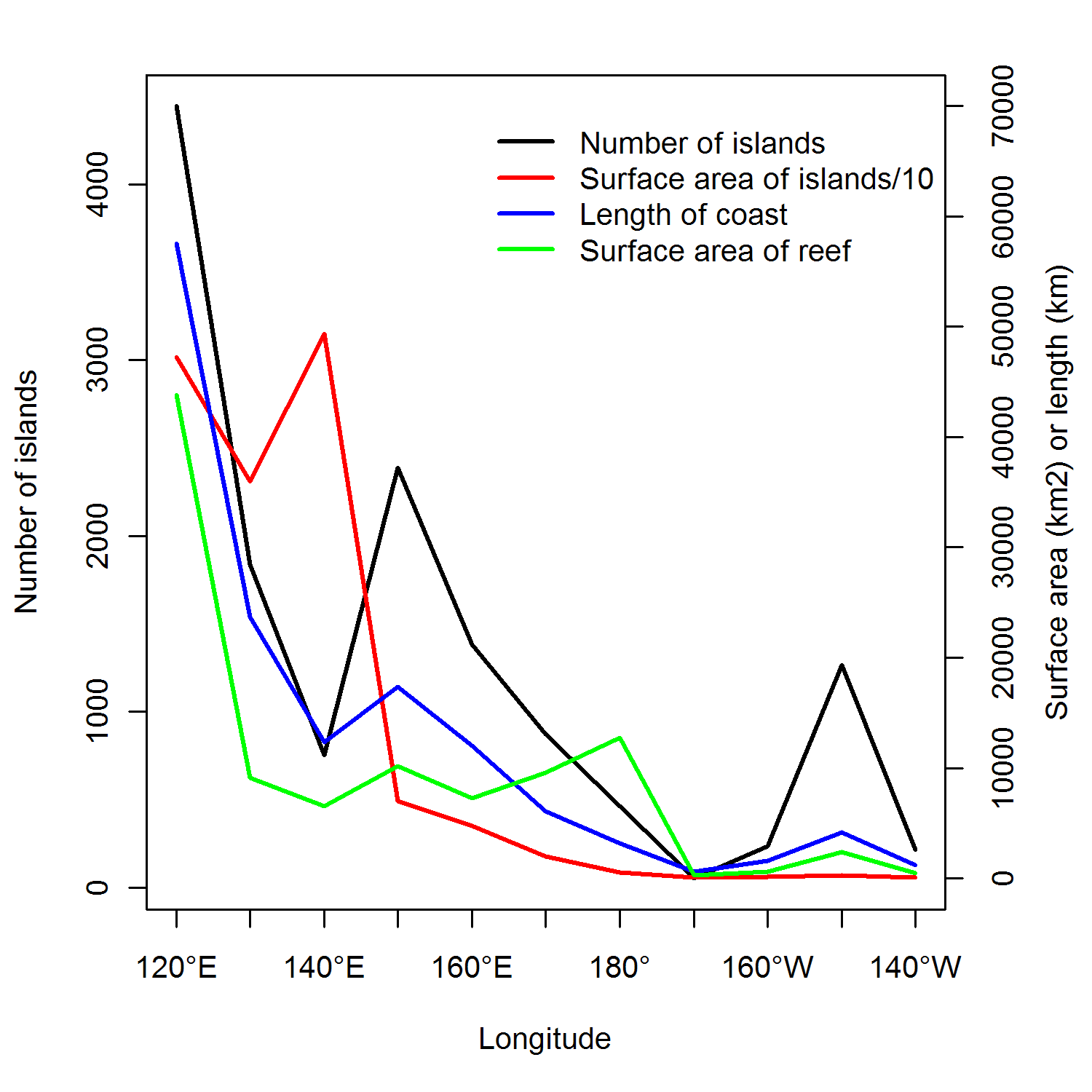

Supplement: Figure S1 — Quantification of island and reef coverage per longitudinal band in the western and central Pacific. Within the 15°N to the 25°S latitudinal band and excluding Australia. (TIF) [file pone.0036701.s001.tif]
