# Supplementary material for: Interaction between Coastal and Oceanic Ecosystems of the Western and Central Pacific Ocean through Predator-Prey Relationship Studies
Source: PLoS One. 2012 May 15;7(5):e36701. doi: 10.1371/journal.pone.0036701 (PMC3352925; doi:10.1371/journal.pone.0036701)
Supplement: Table S1 — Species composition, raw value and percentages of the cumulated weight (W), number (N) and frequency (F) of the reef preys in the diet of the main pelagic predators. (DOCX) [file pone.0036701.s002.docx]

**Table S1.**

| Classification | | | Family | Taxa | W | N | F | %W | %N | %F |
| --- | --- | --- | --- | --- | --- | --- | --- | --- | --- | --- |
| CRUSTACEA | | |  |  |  |  |  |  |  |  |
|  | Decapoda | |  |  |  |  |  |  |  |  |
|  |  | Anomura | |  |  |  |  |  |  |  |
|  |  |  |  | Paguroidea | 0.5 | 5 | 5 | 3.20E-06 | 4.63E-05 | 1.17E-03 |
|  |  |  |  | Anomura zoea | 1.3 | 6 | 5 | 8.32E-06 | 5.56E-05 | 1.17E-03 |
|  |  |  | Paguridae | Paguridae | 19.4 | 177 | 73 | 1.24E-04 | 1.64E-03 | 1.70E-02 |
|  |  |  | Porcellanidae | Porcellanidae | 1.3 | 18 | 12 | 8.32E-06 | 1.67E-04 | 2.80E-03 |
|  |  | Brachyura | |  |  |  |  |  |  |  |
|  |  |  |  | Brachyuran megalopa | 592.4 | 5563 | 1293 | 3.79E-03 | 5.15E-02 | 3.02E-01 |
|  |  |  |  | Brachyuran zoea | 18.8 | 278 | 109 | 1.20E-04 | 2.58E-03 | 2.54E-02 |
|  |  | Caridea | |  |  |  |  |  |  |  |
|  |  |  |  | Alpheoidea | 0.2 | 3 | 1 | 1.28E-06 | 2.78E-05 | 2.33E-04 |
|  |  |  | Alpheidae | Alpheidae | 0.5 | 3 | 2 | 3.20E-06 | 2.78E-05 | 4.67E-04 |
|  |  | Palinura | |  |  |  |  |  |  |  |
|  |  |  |  | Palinuroidea | 74.1 | 189 | 102 | 4.74E-04 | 1.75E-03 | 2.38E-02 |
|  |  |  | Palinuridae | Palinuridae | 38.2 | 61 | 51 | 2.44E-04 | 5.65E-04 | 1.19E-02 |
|  |  |  | Palinuridae | Panulirus sp. | 1.6 | 5 | 5 | 1.02E-05 | 4.63E-05 | 1.17E-03 |
|  |  |  | Palinuridae | Puerulus angulatus | 4.6 | 5 | 3 | 2.94E-05 | 4.63E-05 | 7.00E-04 |
|  |  |  | Palinuridae | Puerulus sp. | 2.5 | 5 | 5 | 1.60E-05 | 4.63E-05 | 1.17E-03 |
|  |  |  | Scyllaridae | Ibacus sp. | 0.9 | 1 | 1 | 5.76E-06 | 9.26E-06 | 2.33E-04 |
|  |  |  | Scyllaridae | Parribacus sp. | 7.2 | 3 | 3 | 4.61E-05 | 2.78E-05 | 7.00E-04 |
|  |  |  | Scyllaridae | Scyllaridae | 12.4 | 26 | 18 | 7.94E-05 | 2.41E-04 | 4.20E-03 |
|  |  |  | Scyllaridae | Scyllarus sp. | 4.7 | 6 | 5 | 3.01E-05 | 5.56E-05 | 1.17E-03 |
|  |  | Thalassinidea | |  |  |  |  |  |  |  |
|  |  |  |  | Thalassinidea | 0.7 | 6 | 3 | 4.48E-06 | 5.56E-05 | 7.00E-04 |
|  | Stomatopoda | | |  |  |  |  |  |  |  |
|  |  |  |  | Stomatopoda | 1077 | 6639 | 2127 | 6.89E-03 | 6.15E-02 | 4.96E-01 |
|  |  |  |  | Squilloidea | 0.7 | 5 | 4 | 4.48E-06 | 4.63E-05 | 9.33E-04 |
|  |  |  | Squillidae | Squillidae | 13.4 | 15 | 10 | 8.58E-05 | 1.39E-04 | 2.33E-03 |
| FISH |  |  |  |  |  |  |  |  |  |  |
|  | Aulopiformes | | |  |  |  |  |  |  |  |
|  |  |  |  | Synodontoidei | 1.1 | 2 | 2 | 7.04E-06 | 1.85E-05 | 4.67E-04 |
|  |  |  | Synodontidae | Synodus sp. | 354.2 | 1073 | 20 | 2.27E-03 | 9.94E-03 | 4.67E-03 |
|  | Beloniformes | | |  |  |  |  |  |  |  |
|  |  |  | Exocoetidae | Parexocoetus sp. | 4 | 1 | 1 | 2.56E-05 | 9.26E-06 | 2.33E-04 |
|  | Beryciformes | | |  |  |  |  |  |  |  |
|  |  |  | Holocentridae | Holocentridae | 122.8 | 97 | 60 | 7.86E-04 | 8.99E-04 | 1.40E-02 |
|  |  |  | Holocentridae | Holocentrinae | 12 | 9 | 6 | 7.68E-05 | 8.34E-05 | 1.40E-03 |
|  |  |  | Holocentridae | Myripristinae | 8.49 | 8 | 2 | 5.43E-05 | 7.41E-05 | 4.67E-04 |
|  |  |  | Holocentridae | Myripristis sp. | 32.2 | 20 | 2 | 2.06E-04 | 1.85E-04 | 4.67E-04 |
|  |  |  | Holocentridae | Sargocentron sp. | 7.7 | 6 | 3 | 4.93E-05 | 5.56E-05 | 7.00E-04 |
|  | Elopiformes | | |  |  |  |  |  |  |  |
|  |  |  | Megalopidae | Megalops sp. | 0.1 | 1 | 1 | 6.40E-07 | 9.26E-06 | 2.33E-04 |
|  | Gasterosteiformes | | |  |  |  |  |  |  |  |
|  |  |  | Fistulariidae | Fistularia commersonii | 28.9 | 1 | 1 | 1.85E-04 | 9.26E-06 | 2.33E-04 |
|  |  |  | Fistulariidae | Fistularia petimba | 4.8 | 2 | 2 | 3.07E-05 | 1.85E-05 | 4.67E-04 |
|  |  |  | Fistulariidae | Fistularia sp. | 6.4 | 3 | 3 | 4.10E-05 | 2.78E-05 | 7.00E-04 |
|  |  |  | Pegasidae | Eurypegasus draconis | 0.4 | 4 | 4 | 2.56E-06 | 3.71E-05 | 9.33E-04 |
|  |  |  | Pegasidae | Eurypegasus sp. | 0.2 | 1 | 1 | 1.28E-06 | 9.26E-06 | 2.33E-04 |
|  |  |  | Syngnathidae | Syngnathidae | 4.5 | 9 | 9 | 2.88E-05 | 8.34E-05 | 2.10E-03 |
|  |  |  | Centriscidae | Centriscidae | 0.1 | 1 | 1 | 6.40E-07 | 9.26E-06 | 2.33E-04 |
|  | Mugiliformes | | |  |  |  |  |  |  |  |
|  |  |  | Mugilidae | Mugilidae | 229.7 | 5 | 3 | 1.47E-03 | 4.63E-05 | 7.00E-04 |
|  | Perciformes | | |  |  |  |  |  |  |  |
|  |  |  | Acanthuridae | Acanthuridae | 1055 | 1543 | 479 | 6.75E-03 | 1.43E-02 | 1.12E-01 |
|  |  |  | Acanthuridae | Acanthurus sp. | 120.6 | 57 | 18 | 7.72E-04 | 5.28E-04 | 4.20E-03 |
|  |  |  | Acanthuridae | Naso sp. | 75.6 | 61 | 25 | 4.84E-04 | 5.65E-04 | 5.83E-03 |
|  |  |  | Siganidae | Siganus sp. | 826 | 486 | 30 | 5.29E-03 | 4.50E-03 | 7.00E-03 |
|  |  |  | Blenniidae | Blenniidae | 11.2 | 53 | 21 | 7.17E-05 | 4.91E-04 | 4.90E-03 |
|  |  |  | Blenniidae | Exallias sp. | 0.2 | 1 | 1 | 1.28E-06 | 9.26E-06 | 2.33E-04 |
|  |  |  | Pomacentridae | Pomacentridae | 0.8 | 4 | 4 | 5.12E-06 | 3.71E-05 | 9.33E-04 |
|  |  |  | Caesionidae | Gymnocaesio gymnoptera | 3.5 | 1 | 1 | 2.24E-05 | 9.26E-06 | 2.33E-04 |
|  |  |  | Carangidae | Atropus atropos | 73.5 | 1 | 1 | 4.70E-04 | 9.26E-06 | 2.33E-04 |
|  |  |  | Carangidae | Decapterus tabl | 13.1 | 1 | 1 | 8.38E-05 | 9.26E-06 | 2.33E-04 |
|  |  |  | Carangidae | Scomberoides sp. | 3.1 | 1 | 1 | 1.98E-05 | 9.26E-06 | 2.33E-04 |
|  |  |  | Carangidae | Selar crumenophthalmus | 32.2 | 4 | 2 | 2.06E-04 | 3.71E-05 | 4.67E-04 |
|  |  |  | Carangidae | Seriola sp. | 45.5 | 1 | 1 | 2.91E-04 | 9.26E-06 | 2.33E-04 |
|  |  |  | Chaetodontidae | Chaetodon sp. | 6.7 | 14 | 8 | 4.29E-05 | 1.30E-04 | 1.87E-03 |
|  |  |  | Chaetodontidae | Chaetodontidae | 130.4 | 293 | 178 | 8.35E-04 | 2.71E-03 | 4.15E-02 |
|  |  |  | Chaetodontidae | Chelmon sp. | 5.9 | 1 | 1 | 3.78E-05 | 9.26E-06 | 2.33E-04 |
|  |  |  | Chaetodontidae | Forcipiger flavissimus | 2.9 | 1 | 1 | 1.86E-05 | 9.26E-06 | 2.33E-04 |
|  |  |  | Chaetodontidae | Forcipiger sp. | 1.6 | 1 | 1 | 1.02E-05 | 9.26E-06 | 2.33E-04 |
|  |  |  | Chaetodontidae | Heniochus sp. | 8.4 | 5 | 5 | 5.38E-05 | 4.63E-05 | 1.17E-03 |
|  |  |  | Kyphosidae | Kyphosidae | 36 | 1 | 1 | 2.30E-04 | 9.26E-06 | 2.33E-04 |
|  |  |  | Kyphosidae | Kyphosus sp. | 2.4 | 1 | 1 | 1.54E-05 | 9.26E-06 | 2.33E-04 |
|  |  |  | Leiognathidae | Leiognathidae | 38.7 | 40 | 6 | 2.48E-04 | 3.71E-04 | 1.40E-03 |
|  |  |  | Leiognathidae | Leiognathus sp. | 30.9 | 35 | 8 | 1.98E-04 | 3.24E-04 | 1.87E-03 |
|  |  |  | Lethrinidae | Lethrinidae | 1.7 | 5 | 4 | 1.09E-05 | 4.63E-05 | 9.33E-04 |
|  |  |  | Lutjanidae | Lutjanus sp. | 14.3 | 40 | 11 | 9.15E-05 | 3.71E-04 | 2.57E-03 |
|  |  |  | Malacanthidae | Hoplolatilus sp. | 19.7 | 23 | 18 | 1.26E-04 | 2.13E-04 | 4.20E-03 |
|  |  |  | Malacanthidae | Malacanthidae | 28.3 | 75 | 49 | 1.81E-04 | 6.95E-04 | 1.14E-02 |
|  |  |  | Malacanthidae | Malacanthus sp. | 1.5 | 1 | 1 | 9.60E-06 | 9.26E-06 | 2.33E-04 |
|  |  |  | Mullidae | Mullidae | 1.1 | 1 | 1 | 7.04E-06 | 9.26E-06 | 2.33E-04 |
|  |  |  | Pomacanthidae | Pomacanthidae | 72.1 | 471 | 169 | 4.61E-04 | 4.36E-03 | 3.94E-02 |
|  |  |  | Priacanthidae | Priacanthidae | 50.3 | 45 | 34 | 3.22E-04 | 4.17E-04 | 7.93E-03 |
|  |  |  | Priacanthidae | Priacanthus sp. | 25.6 | 13 | 5 | 1.64E-04 | 1.20E-04 | 1.17E-03 |
|  |  |  | Serranidae | Cephalopholis sp. | 0.1 | 13 | 1 | 6.40E-07 | 1.20E-04 | 2.33E-04 |
|  |  |  | Serranidae | Serranidae | 10 | 35 | 22 | 6.40E-05 | 3.24E-04 | 5.13E-03 |
|  |  |  | Sphyraenidae | Sphyraena sp. | 104.1 | 1 | 1 | 6.66E-04 | 9.26E-06 | 2.33E-04 |
|  |  |  | Scombridae | Grammatorcynus bilineatus | 12.3 | 1 | 1 | 7.87E-05 | 9.26E-06 | 2.33E-04 |
|  |  |  | Scombridae | Rastrelliger kanagurta | 175.5 | 4 | 2 | 1.12E-03 | 3.71E-05 | 4.67E-04 |
|  |  |  | Zanclidae | Zanclidae | 28.3 | 5 | 4 | 1.81E-04 | 4.63E-05 | 9.33E-04 |
|  |  |  | Zanclidae | Zanclus cornutus | 34 | 4 | 2 | 2.18E-04 | 3.71E-05 | 4.67E-04 |
|  | Scorpaeniformes | | |  |  |  |  |  |  |  |
|  |  |  | Aploactinidae | Aploactinidae | 0.1 | 1 | 1 | 6.40E-07 | 9.26E-06 | 2.33E-04 |
|  |  |  | Scorpaenidae | Pterois sp. | 7.4 | 5 | 5 | 4.74E-05 | 4.63E-05 | 1.17E-03 |
|  | Tetraodontiformes | | |  |  |  |  |  |  |  |
|  |  |  |  | Balistidae/Monacanthidae | 70.5 | 195 | 96 | 4.51E-04 | 1.81E-03 | 2.24E-02 |
|  |  |  | Balistidae | Balistapus sp. | 11.5 | 7 | 5 | 7.36E-05 | 6.49E-05 | 1.17E-03 |
|  |  |  | Balistidae | Balistidae | 1375 | 1556 | 695 | 8.80E-03 | 1.44E-02 | 1.62E-01 |
|  |  |  | Balistidae | Canthidermis sp. | 13.6 | 1 | 1 | 8.70E-05 | 9.26E-06 | 2.33E-04 |
|  |  |  | Balistidae | Rhinecanthus sp. | 72.7 | 25 | 5 | 4.65E-04 | 2.32E-04 | 1.17E-03 |
|  |  |  | Balistidae | Xanthichthys sp. | 15.3 | 1 | 1 | 9.79E-05 | 9.26E-06 | 2.33E-04 |
|  |  |  | Diodontidae | Cyclichthys sp. | 38.9 | 1 | 1 | 2.49E-04 | 9.26E-06 | 2.33E-04 |
|  |  |  | Diodontidae | Cyclichthys spilostylus | 70.7 | 2 | 2 | 4.52E-04 | 1.85E-05 | 4.67E-04 |
|  |  |  | Diodontidae | Diodon sp. | 461.8 | 21 | 23 | 2.96E-03 | 1.95E-04 | 5.37E-03 |
|  |  |  | Diodontidae | Diodontidae | 132.3 | 34 | 30 | 8.47E-04 | 3.15E-04 | 7.00E-03 |
|  |  |  | Monacanthidae | Aluterus sp. | 0.8 | 7 | 2 | 5.12E-06 | 6.49E-05 | 4.67E-04 |
|  |  |  | Monacanthidae | Cantherhines sp. | 31.3 | 4 | 3 | 2.00E-04 | 3.71E-05 | 7.00E-04 |
|  |  |  | Monacanthidae | Monacanthidae | 143.4 | 107 | 62 | 9.18E-04 | 9.91E-04 | 1.45E-02 |
|  |  |  | Monacanthidae | Pervagor sp. | 4.7 | 2 | 2 | 3.01E-05 | 1.85E-05 | 4.67E-04 |
|  |  |  | Monacanthidae | Pseudalutarius nasicornis | 2 | 3 | 2 | 1.28E-05 | 2.78E-05 | 4.67E-04 |
|  |  |  | Ostraciidae | Ostracion sp. | 7.2 | 4 | 4 | 4.61E-05 | 3.71E-05 | 9.33E-04 |
|  |  |  | Ostraciidae | Lactoria diaphana | 390.4 | 26 | 20 | 2.50E-03 | 2.41E-04 | 4.67E-03 |
|  |  |  | Ostraciidae | Lactoria fornasini | 5.4 | 6 | 5 | 3.46E-05 | 5.56E-05 | 1.17E-03 |
|  |  |  | Ostraciidae | Lactoria sp. | 0.5 | 3 | 3 | 3.20E-06 | 2.78E-05 | 7.00E-04 |
|  |  |  | Tetraodontidae | Canthigaster epilampra | 1.7 | 1 | 1 | 1.09E-05 | 9.26E-06 | 2.33E-04 |
|  |  |  | Tetraodontidae | Canthigaster solandri | 1.8 | 2 | 2 | 1.15E-05 | 1.85E-05 | 4.67E-04 |
|  |  |  | Tetraodontidae | Canthigaster sp. | 21.1 | 20 | 3 | 1.35E-04 | 1.85E-04 | 7.00E-04 |
| MOLLUSC | |  |  |  |  |  |  |  |  |  |
|  | Octopoda | |  |  |  |  |  |  |  |  |
|  |  |  | Octopodidae | Octopus defilippi | 0.4 | 2 | 2 | 2.56E-06 | 1.85E-05 | 4.67E-04 |
|  |  |  | Octopodidae | Octopus sp. | 2.5 | 3 | 3 | 1.60E-05 | 2.78E-05 | 7.00E-04 |
|  | Sepiolida | |  |  |  |  |  |  |  |  |
|  |  |  | Sepiolidae | Euprymna sp. | 1.3 | 2 | 2 | 8.32E-06 | 1.85E-05 | 4.67E-04 |
|  |  |  | Sepiolidae | Euprymna tasmanica | 1 | 1 | 1 | 6.40E-06 | 9.26E-06 | 2.33E-04 |

Weight and number are cumulated for all predators and %W, %N and %F are calculated for the 4286 non-empty stomachs cumulated (total cumulated weight of reef and non-reef preys=156153.08g; Total cumulated number of reef and non-reef preys=107941).
