# Supplementary material for: Interaction between Coastal and Oceanic Ecosystems of the Western and Central Pacific Ocean through Predator-Prey Relationship Studies
Source: PLoS One. 2012 May 15;7(5):e36701. doi: 10.1371/journal.pone.0036701 (PMC3352925; doi:10.1371/journal.pone.0036701)
Supplement: Table S2 — Results of the five second-best models of reef prey occurrence in stomach content. (DOCX) [file pone.0036701.s003.docx]

**Table S2.**

|  | BIC | Df | Chisq | p-value |
| --- | --- | --- | --- | --- |
| ~ predator +pred_L+ns(longitude,df=2)+ gear +log(dist_land+1) +(1\|set_code) | 4644 |  |  |  |
| predator |  | 7 | 396.1 | <2.2e-16 *** |
| log(dist_land+1) |  | 1 | 62.6 | 2.52e-15 *** |
| ns(longitude, df=2) |  | 2 | 33.7 | 4.7e-08 *** |
| predator_length |  | 1 | 17.1 | 3.5e-05 *** |
| gear |  | 1 | 10.4 | 0.0013 ** |
|  |  |  |  |  |
| ~ predator + ns(longitude,df=2)+ log(dist_land+1) +(1\|set_code) | 4645 |  |  |  |
| predator |  | 7 | 398.7 | <2.2e-16 *** |
| log(dist_land+1) |  | 1 | 61.4 | 4.8e-15 *** |
| ns(longitude, df=2) |  | 2 | 40.8 | 1.4e-09 *** |
|  |  |  |  |  |
| ~ predator +pred_L+ns(longitude,df=2)+log(dist_land+1)+(1\|set_code) | 4645 |  |  |  |
| predator |  | 7 | 401.7 | <2.2e-16 *** |
| log(dist_land+1) |  | 1 | 54.7 | 1.4e-13 *** |
| ns(longitude, df=2) |  | 2 | 25.7 | 2.6e-06 *** |
| predator_length |  | 1 | 8.3 | 0.004 ** |
|  |  |  |  |  |
| ~ predator +ns(longitude,df=2)+ gear +log(dist_land+1)+(1\|set_code) | 4652 |  |  |  |
| predator |  | 7 | 389.7 | <2.2e-16 *** |
| log(dist_land+1) |  | 1 | 61.3 | 4.1e-15 *** |
| ns(longitude, df=2) |  | 2 | 39.2 | 3.0e-19 *** |
| gear |  | 1 | 1.7 | 0.189 |
|  |  |  |  |  |
| ~ predator +pred_L+ school +ns(longitude,df=2)+log(dist_land+1)+(1\|set_code) | 4652 |  |  |  |
| predator |  | 7 | 401.7 | <2.2e-16 *** |
| log(dist_land+1) |  | 1 | 53.4 | 2.7e-13 *** |
| ns(longitude, df=2) |  | 2 | 27.6 | 1.0e-06 *** |
| predator_length |  | 1 | 10.3 | 0.001 ** |
| school |  | 1 | 2.0 | 0.16 |
|  |  |  |  |  |
| ~ predator + pred_L + school + ns(longitude,df=2) + gear + log(dist_land+1) + (1\|set_code) | 4652 |  |  |  |
| predator |  | 9 | 393.9 | <2.2e-16 *** |
| log(dist_land+1) |  | 1 | 60.9 | 6.0e-15 *** |
| ns(longitude, df=2) |  | 2 | 32.6 | 8.2e-08 *** |
| predator_length |  | 1 | 16.6 | 4.6e-05 *** |
| gear |  | 1 | 8.4 | 0.004 ** |
| school |  | 1 | 0.15 | 0.694 |

See Table 1 legend for details.
