# Supplementary material for: Interaction between Coastal and Oceanic Ecosystems of the Western and Central Pacific Ocean through Predator-Prey Relationship Studies
Source: PLoS One. 2012 May 15;7(5):e36701. doi: 10.1371/journal.pone.0036701 (PMC3352925; doi:10.1371/journal.pone.0036701)
Supplement: Table S3 — Results of the five best models of reef prey proportion in stomach containing reef prey. (DOCX) [file pone.0036701.s004.docx]

**Table S3.**

|  | BIC | Df | Chisq | p-value |
| --- | --- | --- | --- | --- |
| ~ gear +(1\|set_code) | 8374 |  |  |  |
| gear |  | 1 | 127.0 | <2.2e-16 *** |
|  |  |  |  |  |
| ~ gear +log(dist_land+1)+(1\|set_code) | 8377 |  |  |  |
| gear |  | 1 | 79.7 | <2.2e-16 *** |
| log(dist_land+1) |  | 1 | 9.1 | 0.002 ** |
|  |  |  |  |  |
| ~ gear +log(dist_reef+1)+(1\|set_code) | 8379 |  |  |  |
| gear |  | 1 | 97.7 | <2.2e-16 *** |
| log(dist_reef+1) |  | 1 | 7.2 | 0.007 ** |
|  |  |  |  |  |
| ~ school+ gear +log(dist_land+1)+(1\|set_code) | 8380 |  |  |  |
| gear |  | 1 | 18.6 | 1.6e-05 *** |
| log(dist_land+1) |  | 1 | 12.6 | 0.0004 *** |
| school |  | 1 | 5.5 | 0.018 * |
|  |  |  |  |  |
| ~ school+ gear +(1\|set_code) | 8381 |  |  |  |
| gear |  | 1 | 45.9 | 1.2e-11 *** |
| school |  | 1 | 2.1 | 0.147 |

See Table 1 legend for details.
