# Supplementary material for: Interaction between Coastal and Oceanic Ecosystems of the Western and Central Pacific Ocean through Predator-Prey Relationship Studies
Source: PLoS One. 2012 May 15;7(5):e36701. doi: 10.1371/journal.pone.0036701 (PMC3352925; doi:10.1371/journal.pone.0036701)
Supplement: Table S4 — Results of the five best models of reef prey proportion in stomach containing reef prey for predators caught with surface fishing gears. (DOCX) [file pone.0036701.s005.docx]

**Table S4.**

|  | BIC | Df | Chisq | p-value |
| --- | --- | --- | --- | --- |
| ~ pred_L +(1\|set_code) | 4875 |  |  |  |
| predator_length |  | 1 | 46.3 | 1.0e-12 *** |
|  |  |  |  |  |
| ~ pred_L+school+(1\|set_code) | 4884 |  |  |  |
| predator_length |  | 1 | 43.2 | 4.9e-11 *** |
| school |  | 1 | 0.01 | 0.9 |
|  |  |  |  |  |
| ~ pred_L+log(dist_reef+1)+(1\|set_code) | 4885 |  |  |  |
| predator_length |  | 1 | 46.6 | 8.5e-12 *** |
| log(dist_reef+1) |  | 1 | 0.58 | 0.4 |
|  |  |  |  |  |
| ~ pred_L+log(dist_land+1)+(1\|set_code) | 4885 |  |  |  |
| predator_length |  | 1 | 46.3 | 1e-11 *** |
| log(dist_land+1) |  | 1 | 0.5 | 0.5 |
|  |  |  |  |  |
| ~ pred_L+predator+(1\|set_code) | 4886 |  |  |  |
| predator_length |  | 1 | 36.9 | 1.2e-09 *** |
| predator |  | 4 | 20.6 | 0.0004 *** |

See Table 1 legend for details.
