# Supplementary material for: Interaction between Coastal and Oceanic Ecosystems of the Western and Central Pacific Ocean through Predator-Prey Relationship Studies
Source: PLoS One. 2012 May 15;7(5):e36701. doi: 10.1371/journal.pone.0036701 (PMC3352925; doi:10.1371/journal.pone.0036701)
Supplement: Table S5 — Results of the five best models of reef prey proportion in stomach containing reef prey for predators caught with longline fishing gear. (DOCX) [file pone.0036701.s006.docx]

**Table S5.**

|  | BIC | Df | Chisq | p-value |
| --- | --- | --- | --- | --- |
| ~ predator +(1\|set_code) | 3238 |  |  |  |
| predator |  | 6 | 16.7 | 1.7e-14 *** |
|  |  |  |  |  |
| ~ predator +ns(longitude,df=2)+(1\|set_code) | 3245 |  |  |  |
| predator |  | 6 | 79.1 | 5.6e-15 *** |
| ns(longitude, df=2) |  | 2 | 4.4 | 0.11 |
|  |  |  |  |  |
| ~ predator +log(dist_land+1)+(1\|set_code) | 3246 |  |  |  |
| predator |  | 6 | 73.7 | 7.0e-14 *** |
| log(dist_land+1) |  | 1 | 1.3 | 0.24 |
|  |  |  |  |  |
| ~ predator +log(dist_reef+1)+(1\|set_code) | 3247 |  |  |  |
| predator |  | 6 | 73.9 | 6.5e-14 *** |
| log(dist_reef+1) |  | 1 | 0.32 | 0.57 |
|  |  |  |  |  |
| ~ predator +ns(latitude,df=2)+(1\|set_code) | 3248 |  |  |  |
| predator |  | 6 | 76.6 | 1.8e-14 *** |
| ns(latitude, df=2) |  | 2 | 2.4 | 0.30 |

See Table 1 legend for details.
