# Supplementary material for: Interaction between Coastal and Oceanic Ecosystems of the Western and Central Pacific Ocean through Predator-Prey Relationship Studies
Source: PLoS One. 2012 May 15;7(5):e36701. doi: 10.1371/journal.pone.0036701 (PMC3352925; doi:10.1371/journal.pone.0036701)
Supplement: Table S6 — Number of non-empty stomachs examined for the 58 species collected. (DOCX) [file pone.0036701.s007.docx]

**Table S6.**

| Group | Scientific name | Common name | Number of samples |
| --- | --- | --- | --- |
| *Main species* | *Acanthocybium solandri* | Wahoo | 171 |
|  | *Alepisaurus ferox* | Long-snouted lancetfish | 132 |
|  | *Coryphaena hippurus* | Mahi-mahi | 154 |
|  | *Elagatis bipinnulata* | Rainbow runner | 144 |
|  | *Katsuwonus pelamis* | Skipjack | 1114 |
|  | *Thunnus alalunga* | Albacore | 472 |
|  | *Thunnus albacares* | Yellowfin | 1598 |
|  | *Thunnus obesus* | Bigeye | 501 |
| Billfishes |  |  | 200 |
|  | *Istiophorus platypterus* | Indo-Pacific sailfish | 17 |
|  | *Kajikia audax* | Striped marlin | 38 |
|  | *Makaira indica* | Black marlin | 7 |
|  | *Makaira nigricans* | Blue marlin | 43 |
|  | *Tetrapturus angustirostris* | Shortbill spearfish | 48 |
|  | *Xiphias gladius* | Swordfish | 47 |
| Sharks |  |  | 57 |
|  | *Carcharhinus albimarginatus* | Silvertip shark | 1 |
|  | *Carcharhinus falciformis* | Silky shark | 25 |
|  | *Carcharhinus leucas* | Bull shark | 1 |
|  | *Carcharhinus longimanus* | Oceanic whitetip shark | 3 |
|  | *Galeocerdo cuvier* | Tiger shark | 1 |
|  | *Isurus oxyrinchus* | Short-finned mako shark | 11 |
|  | *Isurus paucus* | Longfin mako shark | 4 |
|  | *Prionace glauca* | Blue shark | 10 |
|  | *Sphyrna lewini* | Scalloped hammerhead shark | 1 |
| Other fishes |  |  | 316 |
|  | *Alepisaurus brevirostris* | Short-snouted lancetfish | 8 |
|  | *Alepisaurus sp.* | Lancetfish | 7 |
|  | *Aluterus monoceros* | Unicorn leatherjacket filefish | 1 |
|  | *Assurger anzac* | Razorback scabbardfish | 1 |
|  | *Auxis thazard* | Frigate tuna | 20 |
|  | Balistidae | Other triggerfishes | 29 |
|  | *Brama brama* | Atlantic pomfret | 2 |
|  | Bramidae | Other pomfrets | 7 |
|  | *Canthidermis maculata* | Ocean triggerfish | 3 |
|  | *Caranx sexfasciatus* | Bigeye trevally | 1 |
|  | Chiasmodontidae | Snaketooth fish | 2 |
|  | *Dasyatis violacea* | Pelagic stingray | 29 |
|  | *Decapterus macarellus* | Mackerel scad | 4 |
|  | *Desmodema polystictum* | Polka-dot ribbonfish | 1 |
|  | *Euthynnus affinis* | Kawakawa | 42 |
|  | Gempylidae | Other gemfish and snake mackerels | 1 |
|  | *Gempylus serpens* | Snake mackerel | 3 |
|  | *Gnathanodon speciosus* | Golden trevally | 4 |
|  | *Lampris guttatus* | Moonfish | 68 |
|  | *Lepidocybium flavobrunneum* | Escolar | 9 |
|  | *Lobotes surinamensis* | Tripletail | 1 |
|  | *Melichthys niger* | Black triggerfish | 2 |
|  | *Mobula japanica* | Spinetail mobula | 2 |
|  | *Omosudis lowii* | Hammerjaw | 2 |
|  | *Platax sp.* | Batfish | 1 |
|  | *Platax teira* | Longfin batfish | 1 |
|  | *Promethichthys prometheus* | Roudi escolar | 2 |
|  | *Ruvettus pretiosus* | Oilfish | 4 |
|  | *Scombrolabrax heterolepis* | Longfin escolar | 5 |
|  | Scopelarchidae | Pearleyes fish | 1 |
|  | *Sphyraena barracuda* | Great barracuda | 37 |
|  | *Sphyraena qenie* | Blackfin barracuda | 1 |
|  | *Sphyraena sp.* | Other barracudas | 6 |
|  | *Taractichthys longipinnis* | Big-scaled pomfret | 4 |
|  | *Taractichthys steindachneri* | Sickle pomfret | 5 |

The analysis was based on the 8 main species with more than 100 samples.
